# Dr. S. O. Gleason

**Published:** 1899-05

**Authors:** 


DR. S. O. GLEASON, of Elmira, died in Buffalo early in April. He established the Elmira Sanitarium about fifty years ago, which of late has taken the name of the founder and is called the Gleason Sanitarium. He was for many years professor of chemistry in the Geneva Medical College, and through his influence women were admitted to that institution. His wife, who survives him, was Dr. Rachael Brooks, and his son, Mr. E. B. Gleason, is business manager of the Gleason sanitarium.

